# From SARS to COVID-19: the role of experience and experts in Hong Kong’s initial policy response to an emerging pandemic

**DOI:** 10.1057/s41599-022-01467-z

**Published:** 2023-01-05

**Authors:** Kira Matus, Naubahar Sharif, Alvin Li, Zhixin Cai, Wai Haang Lee, Max Song

**Affiliations:** Hong Kong University of Science and Technology, Hong Kong, China

**Keywords:** Science, technology and society, Politics and international relations

## Abstract

As one of the most densely populated places in the world, Hong Kong fared relatively well in the first year of the COVID-19 pandemic, with a very low number of cases and fatalities per capita. This was mostly due to the Hong Kong government, healthcare workers, and the general public’s institutional and individual memory after they successfully overcame the deadly severe acute respiratory syndrome (SARS) epidemic in 2003. However, while Hong Kong was well accustomed to measures such as wearing masks and social distancing, the cooperation of the Hong Kong public to government restrictions was highly affected by its local political context, especially after widespread anti-government protests began mid-2019. This brought the public’s trust in government to an all-time low, creating a political ‘new normal’, which underpinned how COVID-19 policies would be proposed, accepted, and implemented, if at all. To understand how science advice was offered and how public health decisions were made, this research investigates the evolution of Hong Kong’s science advisory mechanisms for public health from before SARS, after SARS, and during COVID-19 in 2020, including the roles of key organisations and departments, the establishment of new centres and committees, and the creation of workgroups and expert advisory panels. This paper compares and analyses the reasons behind these differences in science advisory mechanisms between SARS and COVID-19. The findings from this research reinforce the unquestionable need for robust science advisory structures and knowledgeable scientific experts to solve health-related crises, though more research is required to understand the ways in which science advice influences both policy decisions and public acceptance of these policies.

## Introduction

In the earliest days of 2020, news stories from Wuhan about an emerging virus outbreak sparked discussion and concern among the residents of Hong Kong (Leung and Chan, 2020). This viral outbreak coincided with a political outbreak characterised by large-scale protests and social division since June 2019 (McLaughlin, 2020). Wuhan, the epicentre of the disease known as COVID-19, caused by the novel SARS-CoV-2 coronavirus, was a short high-speed rail ride away from Hong Kong. By Chinese New Year, which was 3 weeks after the first confirmed cases, Wuhan and other parts of Mainland China were in lockdown, and it was clear that COVID-19 would disrupt daily life for the Hong Kong locals. As the threatening evolution from sporadic outbreaks to a widespread epidemic was appearing serious and imminent, both the government and the public were preparing to respond. By the third week of January, the Hong Kong Special Administrative Region’s (HKSAR) government had activated its anti-epidemic response plans. Concurrently, the people of Hong Kong—who still had deep memories of the severe acute respiratory syndrome (SARS) epidemic in 2003—responded promptly and willingly with high rates of voluntary masking, hand-washing, social distancing, and the use of thermometers at the entrance of many public spaces (Chow, 2020). Despite the implementation of such containment strategies, the virus slipped through the borders and spread across continents to become a global pandemic.

### Hong Kong’s initial strong performance for COVID-19

Many local residents felt that the government had missed opportunities to make swifter and better decisions, such as the delayed decision to close the border with the neighbouring Mainland China (Wu, 2020). However, Hong Kong weathered 2020 as one of the better-performing territories, despite its status as Asia’s hub for international travel and trade (Yuen et al., 2021). As of 31 December 2020, Hong Kong had logged 8846 confirmed cases and 148 deaths, in a city of ~7.5 million people—1.37 million of whom were over 65, the age group with the highest mortality rate (Ritchie et al., 2022; see Supplementary Information Appendix 1 for case figures). Hong Kong had managed to keep the rate of infection low without ever resorting to full, formal lockdowns. Instead, the government had implemented a series of policies, including travel restrictions, testing and contact tracing, quarantines and isolations, school closures, restrictions on public gatherings and public activities (including dining hours), and mask-wearing mandates (Chan et al., 2021). Some of these policies were eagerly accepted, while others resulted in controversy, resistance, and non-compliance. Through it all, the government’s announcements were accompanied by advice and comments from a variety of advisory committees and scientific experts who make up Hong Kong’s science advisory system for COVID-19.

### SARS and its impact on Hong Kong

Hong Kong’s COVID-19 science advisory system and its pandemic responses should be understood with special consideration of two contextual perspectives: historical and political. The first case of SARS, a highly infectious and life-threatening viral respiratory disease, first appeared in Southern China in November 2002 and subsequently appeared in Hong Kong in February 2003 (World Health Organization, 2022). The virus caused patients to exhibit major clinical features such as persistent fever, malaise, chills, and dry cough (Hui et al., 2003), yet, clinicians had no experience in or effective treatment options to eliminating the virus beyond symptomatic relief and immunological support (Stockman et al., 2006). The virus spread beyond Mainland China and Hong Kong’s borders via international travels to 29 countries and territories, reaching Taiwan, Singapore, Canada and Australia, though most cases remained within Asia (Chan-Yeung and Xu, 2003). Owing to the virus’s short incubation period of 2–10 days and high case fatality rate of up to 12% (Sampathkumar et al., 2003)—characteristics that allowed for early detection and isolation and therefore the severance of community transmission chains—the SARS epidemic had largely subsided by June 2003 after bringing a total of 8096 cases and 774 deaths worldwide (World Health Organization, 2015), short of evolving into an out-of-control, fully fledged global pandemic like COVID-19.

In the wake of SARS crossing the borders into Hong Kong, local officials instituted a series of public health reforms, including the formation of the Center for Health Protection (CHP; Hospital Authority Ordinance, 1997). The CHP comprises a number of standing expert committees, and has been developing response plans and conducting cross-government drills, all designed to improve the ability of Hong Kong to respond to health emergencies, including novel epidemics and pandemics (Center for Health Protection, 2005). This system led by CHP was in place and activated early in 2020 in response to COVID-19. The population’s collective memory of SARS also positively impacted their understanding of and response to COVID-19; people were well accustomed to wearing masks, and public schools were experienced in closing for short periods in previous years with disruptive influenza outbreaks (Cowling et al., 2020). They had largely respected—and in some cases, enthusiastically embraced—mask-wearing, hand-washing, and other official and unofficial rules and norms to support social distancing and other preventative measures.

The second perspective to examine is the local political context and circumstances, which went on to determine the overall acceptance by and cooperation among the Hong Kong public, despite the pre-learned behaviours from SARS. In 1984, the Chinese and British governments signed the Sino-British Joint Declaration, an international treaty, which outlined the mutually agreed terms of the ‘handover’ of sovereignty from the United Kingdom to China in 1997. The Joint Declaration also declares the ‘one country, two systems’ principle, along with the Basic Law (Hong Kong’s constitutional document) both stipulating that Hong Kong’s economic system and social way of life would be unchanged for fifty years until 2047 (HKSAR Government, 2021a). The Basic Law designates a system of governance led by the Chief Executive and an Executive Council. Before the Chief Executive makes important policy decisions or introduces bills and budgets to the legislature, he or she shall consult the Executive Council—whose 32 members are appointed by the Chief Executive—except when adopting certain measures in emergencies. Separately, the Legislative Council (LegCo) is the elected law-making body, who, on top of law-making duties, debate, scrutinise, and vote on budgets and laws, including those proposed by the Chief Executive. The rest of HKSAR’s civil service conducts the administrative and executive functions of the government and employs 4.4 per cent of Hong Kong’s workforce (174,900 people), spanning 13 policy bureaux and 56 departments (HKSAR Government, 2021b).

Ever since the handover in 1997, Hong Kong people have regularly protested against various proposals to alter their freedoms and rights. A demonstration in 2003 protested the decline in freedom of speech (said to be limited by the enactment of the Article 23 of the Basic Law, which would create Hong Kong’s own national security law) and the ‘Occupy’ movement in 2014 protested the ‘brainwashing’ of values (said to be caused by a proposed”national education” system; Gunia, 2019). In June 2019, the Hong Kong government was due to pass the Fugitive Offenders and Mutual Legal Assistance in Criminal Matters Legislation (Amendment) Bill 2019 (more commonly known as the “extradition bill”), which would allow extradition of criminals to Mainland China. This triggered widespread protests in fear that this bill would undermine judicial independence and violate the freedoms that Hong Kong had enjoyed thus far (BBC, 2019). At the height of the movement, almost two million people (~25% of Hong Kong’s population) reportedly marched on the streets (SCMP Reporters, 2019) calling for the withdrawal of the extradition bill, the implementation of universal suffrage, as promised in the Basic Law, and the stepping down of Chief Executive Carrie Lam, among other demands.

After months of clashes with pro-Beijing government officials, legislators, and a hardline police force, trust in government hit an all-time low. A survey conducted in February 2020 by Hong Kong Public Opinion Research Institute (2020) revealed that trust in the HKSAR government had fallen to 14%, while distrust in the government rose to an all-time high of 76%. The proposed bill itself, accompanied by the government’s strong responses to the protests, have caused extreme polarisation of political ideologies; one side continued to call for electoral reform and democratic rights, while the other supported the government and police in using authority to ensure economic and social stability (Shen and Yu, 2021). This months-long crisis, just months prior to the beginning of the COVID-19 pandemic, had created a political ‘new normal’ for Hong Kong that involved record low levels of public trust in the government and the politicisation of policies that were relatively apolitical, and underpinned how COVID-19 policies would be proposed, implemented or accepted (Hartley and Jarvis, 2020). This later proved to be a challenge for the Hong Kong government when implementing more stringent and controversial anti-epidemic policies in the year 2020.

### Methodology

This research takes the form of an descriptive, in-depth case study that conducts comparative analysis of the two pandemics within one political context. The aim of such study is to draw parallels and contrasts in the policy responses, scientific advisory mechanisms, communications and management between the two pandemics, as experienced in Hong Kong. Some of the broad research questions include: How did Hong Kong’s experience of the SARS crisis fundamentally affect public health structures in preparation for future pandemics? Were the measures and structures in place during SARS and post-SARS sufficient for handling COVID-19? How, and why, was pandemic response different between SARS and COVID-19? Despite the expected opacity in the governmental decision-making processes that occur behind-the-scenes, the inclusion of a wide range of publicly available archival documents will paint a clear and complete picture of Hong Kong’s public health mechanisms, both in the past and in the present.

This single, historical case study provides an institutionally focused account of the differences and changes in policy response in Hong Kong between its two most prominent pandemics: SARS and COVID-19. It is based largely on an analysis of policy documents, government statistics, media accounts and academic literature. For SARS, brochures and documents that were self-published by various governmental departments were used to assess the purpose and functions of advisory structures (e.g., Center for Health Protection, 2005). Guidelines and checklists used to instruct operational stakeholders like policy decision makers, hospital managers, frontline health workers, and ‘cleansing’ operators, were used to appraise the resources and processes required to deliver the intended outputs and outcomes (e.g., HKSAR Government, 2003a). A review of the lessons learned from the SARS epidemics based on the academic literature already published that evaluated governmental response to SARS and made recommendations for future policy change (e.g., Lee, 2003; SARS Expert Committee, 2003a).

The source of the official archival documents for COVID-19, in contrast, was more centralised; due to the long-lasting nature of COVID-19 and a general increase in internet and digital media use, the relevant documents, notices, updates and guidance distributed by the government were accessible through a dedicated, one-stop “COVID-19 Thematic Website” launched and revamped in February 2020 (HKSAR Government, 2020h). This centralised website links to multiple other official departments’ and press conferences directories, such as Center for Health Protection and GovHK. Given the rise in the popular use of digital, mobile and social media for COVID-19 information dissemination (Bao et al., 2020), much of governmental communication to the press and the public were channelled through press conferences over live stream platforms (e.g., on Facebook, online TV channels), as well as updating the public on pandemic developments and case numbers via social media platforms (e.g., Instagram). Using these sources of information, this research was able to track, in real time, the government’s policy responses and any changes or updates to scientific advisory mechanisms, such as the appointment of scientific experts on vaccinations.

### Science advice during crises

Theoretically, policy responses to pandemics can be seen as a response to an emerging *crisis*. From the literature of crisis management, a crisis can be defined as an event during which an urgent threat to the structures, core values and functions of a system—as perceived and experienced by a government, organisations, communities and the wider population—requires making vital decisions under conditions of time pressure and high uncertainty (Rosenthal and Kouzmin, 1997; Rosenthal et al., 2001; Boin and ‘t Hart, 2007). The “context of the disaster” can occasionally be defined and determined, too, by the mass media and its narratives (Rosenthal et al., 2001). While having a narrow, exclusionary definition of a ‘crisis’ is unproductive for theoretical development (Pursiainen, 2022), in reality, most would agree to characterise SARS and COVID-19 as a crisis requiring critical crisis management and crisis communication (Wodak, 2021). Definitionally, these pandemics threatened core values (e.g., safety, security, health, fairness), created a sense of urgency (e.g., due to the need to swiftly isolate the infected and stop transmission chains) and exhibited a high degree of uncertainty (e.g., in transmission, symptoms, treatment and mutated variants; ‘t Hart and Tummers (2019)). Specifically, the pandemics encompassed two types of crises using ‘t Hart’s (2014) typology: a situational crisis, where disruptive and unexpected incidents occur ‘out there’ (e.g., the virus spreading in the community, healthcare systems in burden), and an institutional crisis, where the problem lies in ineffective governments, inefficient organisations or politicised policies. While these types of crisis can exist in silo, each type of crisis can trigger the other type; for instance, an unaddressed situational crisis could ‘metastasise’ into a serious institutional crisis (Petridou et al., 2020).

How does crisis management materialise as a form of policymaking? Pandemic response can be viewed in the perspective of the crisis management cycle posited by Drennan et al. (2014). Prior to SARS or COVID-19, public health systems would be in the *preparation* phase, conducting simulations, training and contingency planning, based on past experiences. As a novel coronavirus emerges, the government and public health agencies would begin the *response* phase, mobilising operational resources to community workers and hospital managers and financial resources to hard-hit industries and low-income or disadvantaged communities. Drawing parallels to policy learning literature, crises open ‘policy windows’ (Kingdon, 1984) that provide opportunities for change and to overcome governmental inertia that often inhibits policy learning under ‘normal’ conditions (Stern, 1997). As the pandemic (or each wave) subsides, the multiple agencies can buy time in the *recovery* phase to debrief, rebuild, enquire and learn valuable lessons for future policymaking (Moynihan, 2008). With this, *prevention* efforts can take place, such as threat assessment and mitigation strategising, before embarking on preparatory work again.

Specific to the Hong Kong context, SARS triggered a strong crisis management cycle to begin at the response phase with little prior preparation, given how deadly, unprecedented and unexpected the epidemic was (Lee, 2003). After SARS, the recovery phase with adequate policy learning occurred for the ensuing months and years, with multiple independent reviews and scholarly evaluations (Lee, 2003). This recovery phase also consolidated the public’s vivid and emotional memories of SARS into lasting public health knowledge and health-seeking behaviours (Lau et al., 2005). The evaluations led to new prevention measures to be taken (detailed in our findings) that subsequently helped to prepare for the epidemics that followed, including avian flu and swine flu. These mild outbreaks occasionally triggered only a minor activation of the crisis management cycle—that is, until COVID-19 hit suddenly, triggering an unprecedented origination of the crisis management cycle once again. The lingering and less deadly nature of COVID-19 also bought scientists and policy-makers time for more hindsight evaluation, research, policy trial-and-error and foresight for supportive measures.

Ultimately, the effectiveness of the relevant policy responses depends largely on whether crisis leadership was exerted; that is, did those with crisis management responsibilities fulfil all the expected and required tasks to facilitate an effective response? Some of these key tasks inherent in successful crisis leadership were characterised by Boin et al. (2013) as sensemaking of the nature of the crisis (the pandemic), orchestrating coordination among organisations (governmental departments and public health agencies), communicating with citizens and cooperative organisations (via the media, appointed experts and industry associations) and honing their own capacity to learn from failure (gathering feedback from social distancing measures). Interestingly, counter to conventional wisdom that crisis management problems arise from poor inter-agency communication and coordination (Quarantelli, 1988), scholars also suggest that such inter-agency tensions may yield positive outcomes, such as counteracting ‘groupthink tendencies’ and promoting openness (Rosenthal et al., 1991). Under the lens of crisis management, the case of Hong Kong’s pandemic response for SARS and COVID-19 will shine a light on the role of crisis experience on policy decision making.

This research focuses on key theoretical contributions pertaining to the role of institutional science advice and scientific experts in crisis management. One core feature of pandemic crises that differs from other crises (like financial crises or conflict), is that policy decisions are led by scientific evidence on the viral threat and up-to-date knowledge in public health. Politicians, policy-makers and economists, alone, do not have sufficient knowledge nor capability to reliably understand or make judgements or decisions on science-based policies, e.g., those related to epidemiology or vaccine risks and efficacy. The findings from this research reinforce the unquestionable need for robust science advisory structures and knowledgeable scientific experts to solve health-related crises, tackling both the situational aspect of the crisis, as well as preventing the institutional aspect. This paper outlines the evolution of Hong Kong’s science advisory structures and use of experts over time in response to two major pandemics, and how pandemic crisis management has resulted in temporary and/or permanent changes to Hong Kong’s public health structures and policymaking.

In this paper, we will analyse in depth the structures and approaches taken by the Hong Kong government and scientific advisors in response to COVID-19 in the year 2020. Section ‘Hong Kong’s science advisory mechanism for public health during the SARS era’ provides a summary of the science advice mechanisms that were in place *during SARS*, as a starting point for understanding the emergence of the more recent structures operating in 2020. Section ‘Hong Kong’s science advisory mechanism in the post-SARS and pre-Covid era’ describes the system that was developed *in the wake of SARS*, and which was operating at the end of 2019 and the start of 2020, when COVID-19 first began to present itself as a problem for Hong Kong. Section ‘Hong Kong’s science advisory mechanism during the COVID-19 pandemic’ focuses on the science advice structure *during COVID-19’s onset in 2020*, which was largely based on the initial structure in place, but with some changes in response to the unfolding pandemic. Section ‘Comparison between the responses to SARS and COVID-19 and related science advisory mechanisms’ compares the differences between the responses to SARS and responses to COVID-19. Section ‘Analysis of the differences between scientific advisory mechanisms for SARS and COVID-19’ analyses the various differences in the science advisory structures during the two pandemics.

## Hong Kong’s science advisory mechanism for public health during the SARS era

**Fig. 1:**
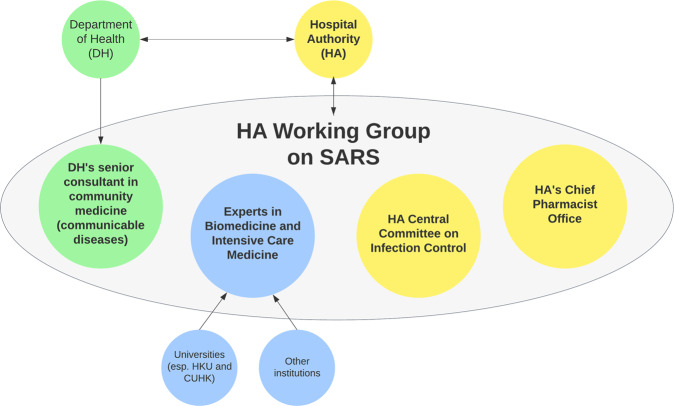
The core decision making and reporting entities in response to SARS. Hong Kong’s scientific advisory board for SARS was established under HA, which managed public hospitals and healthcare provision, instead of under DH, which communicated and coordinated with the local government and industry groups, as well as Mainland Chinese and international organisations.

In early 2003, the SARS epidemic posed an unprecedented challenge to the Hong Kong government’s capability in responding to public health emergencies. The rapid progression of SARS and the immense pressure placed on the healthcare system and the entire socio-economy exposed the government’s areas of weakness, and emphasised the critical role of scientific advisory mechanisms in supporting decision-making during crisis times (Lee, 2003). The Department of Health (DH) had not, at the time, established a dedicated scientific advisory body. Instead, the DH and the Hospital Authority (HA) played the major role in pandemic responses (see Fig. 1). Officially, DH’s responsibilities can be summarised as follows (Department of Health, 2022):

- Making major public health decisions together with the Chief Executive in response to SARS;
- Leading and coordinating the investigation and contact tracing of major cluster infection cases, treatment capacity building, in-hospital infection control and community public education, in collaboration with HA;
- Conducting collaboration and coordination with other governmental departments, various industry associations, communities and other social organisations;
- Maintaining liaison and coordination with other entities outside of Hong Kong, including the central government in the mainland (Ministry of Health), Guangdong Province and related cities, WHO and government agencies in other countries, such as the United States.

Compared to the DH, the HA did not take on much of an internal or external government communication role, but instead focused on regulating and managing public hospitals and supporting and implementing public health decisions in collaboration with the DH (Hospital Authority, 2003; 2022b). Officially, the HA’s responsibilities included:

- Leading hospitals in the screening, isolation, and treatment of SARS cases, as well as preventing and controlling intra-hospital infections;
- Conducting investigation and contact tracing of major cluster infection cases, capacity building of treatments, in-hospital infection control, and community public education, in collaboration with DH;
- Providing scientific advice through participating in an expert working group on the treatment of SARS and the prevention and control of the outbreak.

This working group was renamed from the initial ‘HA Working Group on Severe Community-Acquired Pneumonia’ to the’HA Working Group on SARS’, and it served as the specific science advisory body in response to the SARS outbreak (LegCo, 2003; Caulfield and Liu, 2006). The decision that hospitals—particularly public and larger private ones—and the relevant governmental public health departments took the lead in the epidemic response was mostly due to the nature of the virus and outbreak. Most notably, SARS had a rapid onset of very severe and visible symptoms that required patients to be immediately hospitalised and in intensive care, while ‘super-spreaders’ had the ability to rapidly spread the virus to large-scale clusters such as tall apartment buildings (Chan-Yeung and Xu, 2003). These characteristics shifted the burden of response to healthcare provision and therapeutics, instead of administrative, political, and socio-economic decisions. This explains why the aforementioned ‘working group’ was established under the HA instead of the DH.

**Fig. 2:**
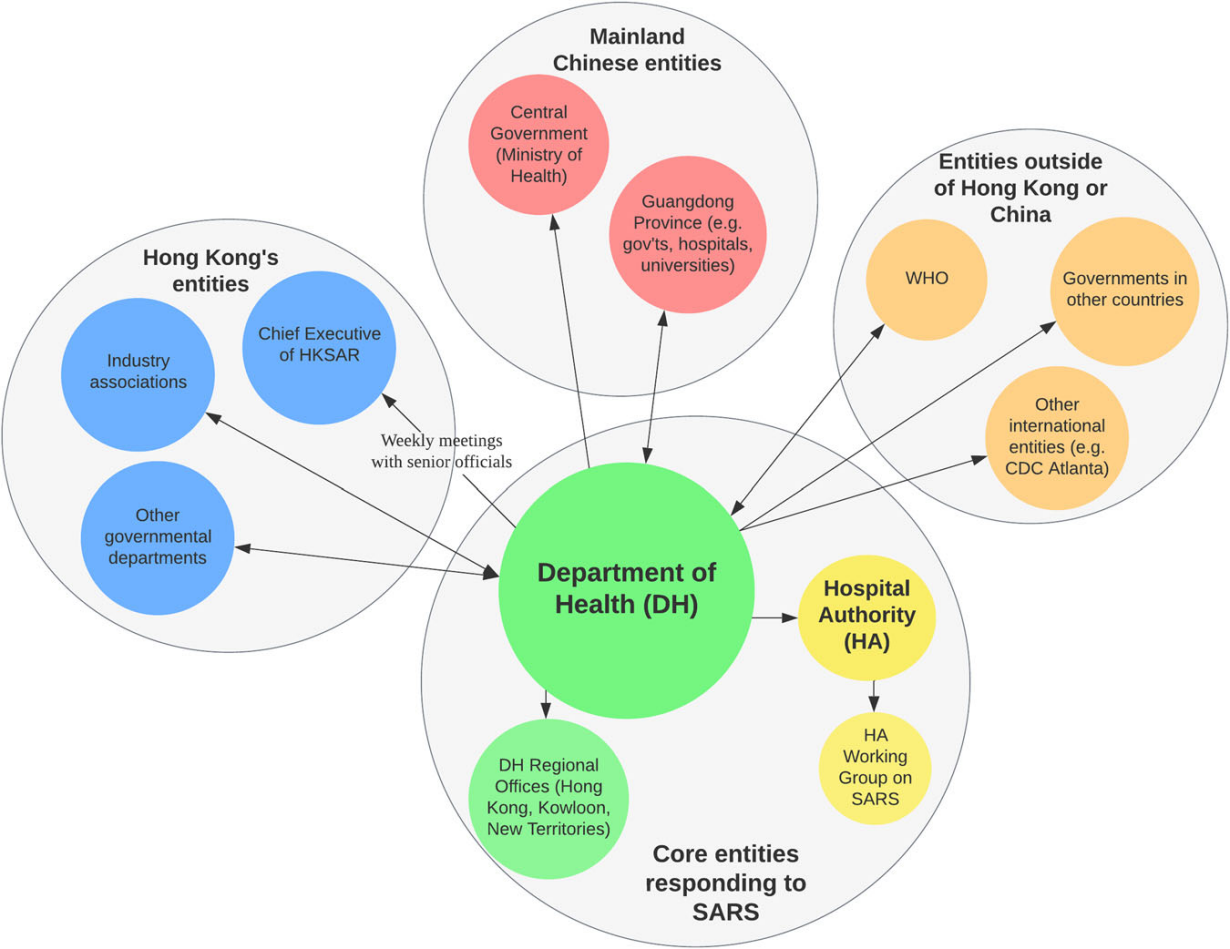
The stakeholders under HA’s Working Group on SARS. The HA’s Working Group on SARS initially included only a small number of clinical experts, but expanded soon after to include many more members from other relevant disciplines, such as community medicine and infectious disease control.

This working group was first established by the HA after the Mainland Chinese central government’s Ministry of Health communicated with Hong Kong’s DH on 11 February 2003 regarding the suspected outbreak of infectious pneumonia in the Guangdong Province (HKSAR Government, 2005a). Initially, this working group included experts in microbiology and intensive care medicine, and intended to offer advice on the monitoring of Severe Community-Acquired Pneumonia outbreaks and the corresponding solutions (see Fig. 2). Six days later, on 17 February, the HA’s working group expanded its membership to include the DH’s senior consultant in community medicine, as well as all members of the HA’s Central Committee on Infectious Disease Control and the Chief Pharmacist Office. The newly structured HA Working Group combined the roles of the DH and the HA, while also involving experts from hospitals, universities, and other institutions, as well as the Chief Pharmacist’s Office, forming a more multidisciplinary SARS-specific scientific advisory mechanism (HKSAR Government, 2005a).

Through the SARS pandemic, the DH, HA and HA’s Working Group on SARS gradually developed a public health decision support mechanism as follows:

- Public hospitals under HA’s jurisdiction would report SARS cases and the local situation in their locale to HA and DH’s regional offices. From February 13 onwards, private hospitals were required to notify DH of any SARS cases.
- HA and DH officials would then work with the hospitals concerned to conduct case investigation and contact tracing.
- Meanwhile, through internal meetings within HA Working Group on SARS, members would evaluate the pandemic situation thus far and make relevant policy recommendations.
- These policy recommendations would be communicated upward through DH and HA to HKSAR’s Chief Executive in the form of weekly meetings with senior officials, who would make the relevant policy decisions. Related information would also be shared by the DH with WHO, Guangdong Province, and the Ministry of Health of the central government.
- Additionally, public health advice regarding various activities (e.g., transport, travel, business, schools, community) would be communicated by DH to other governmental departments and industry associations.

In this decision support mechanism, the investigative work of the DH and HA would form the ‘input’ of information, which would then be processed through scientific advisory meetings by HA Working Group on SARS and become the reference for decision making (Center for Health Protection, 2005). These references would then be passed upwards to the Chief Executive to become the final ‘output’ or the ‘decision’, which would then be transpired down and implemented through the DH, HA, other governmental departments, communities and industry associations. Through the SARS epidemic, the Hong Kong government began to finally establish a scientific advisory mechanism specific to supporting public health, which would eventually form the basis of the government’s public health scientific advisory body used for epidemics in the post-SARS future.

## Hong Kong’s science advisory mechanism in the post-SARS and pre-Covid era

**Fig. 3:**
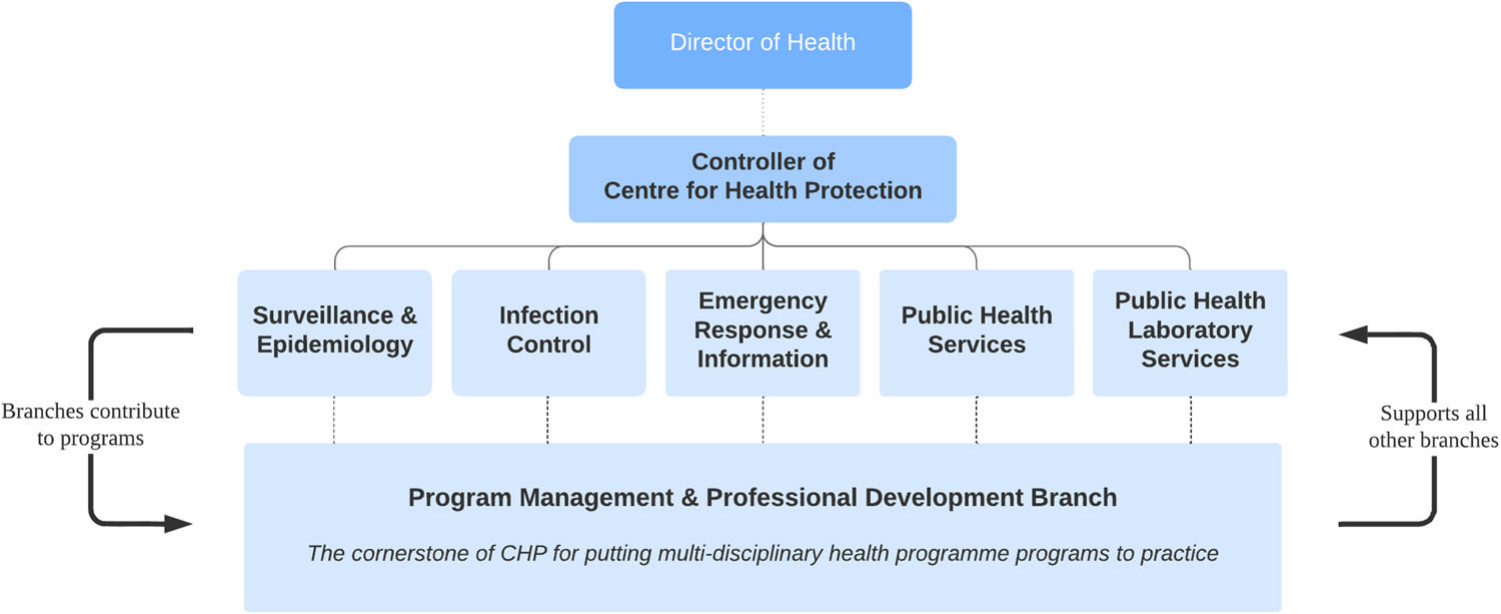
The six branches of CHP. Each of CHP’s six branches provide different services, yet they collaboratively and collectively serve the wider purpose of public health protection.

After SARS, the Hong Kong government set up a SARS Expert Committee to review the recent epidemic (Hong Kong Government, 2003c; SARS Expert Committee, 2003a). This committee’s main recommendation was to set up a body under the DH to research and prevent diseases, and this led to the establishment of the Center for Health Protection (CHP) in 2004 (SARS Expert Committee, 2003b). In order to create a comprehensive, holistic and multidisciplinary health protection system, these SARS Expert Committee members were selected according to their wide range of expertise, ranging from hospital management and public health to epidemiology and respiratory disease (SARS Expert Committee, 2003d; see Supplementary Information Appendix 2 for members of the SARS Expert Committee). While the CHP comprises six different branches that manage different areas of public health (detailed below; see Fig. 3), they leverage their collective knowledge and resources to respond to emerging public health threats and issues (Center for Health Protection, 2004c).

Since its establishment, the CHP as played a crucial role in leading the prevention of infectious diseases and eventually became the main science advisory body around public health issues (Center for Health Protection, 2022). The duties of CHP are to establish a disease surveillance system, strengthen infection control, enhance laboratory diagnostic capacity, conduct risk communication and health promotion, develop applied research and training programmes, and prepare emergency response plans (Lam, 2007).

There are six functional branches under the CHP shouldering different health protection programmes, as follows:

1. The **Surveillance and Epidemiology Branch** was built for facilitating and extending the surveillance on new outbreaks of diseases in the community as well as epidemiological investigation and control for both communicable and non-communicable diseases. From these investigations, this branch would formulate strategies and action plans to be implemented. They also act as a networking system with the DH, HA and other health care professionals and parties.
2. The **Emergency Response and Information Branch (ERIB)** is responsible for preparing for emergency situations, managing health crises and updating contingency plans. The branch organises drills regularly to improve risk communication strategies between departments within the government (see Supplementary Information Appendix 3 for examples of past drills).
3. The **Infection Control Branch** devises and updates guidelines and protocols for medical staff on infection controls. They run training workshops for non-hospital staff with the most up-to-date methods of protection.
4. The **Public Health Services Branch** manages efforts for disease prevention, with a focus on tuberculosis, HIV, and sexually transmitted infections. This branch also provides specialised services and strategy development for these communicable diseases.
5. The **Public Health Laboratory Services Branch** provides laboratory diagnostic services for disease surveillance and control, health promotion and disease prevention, public health consultation service relating to microbiology and virology, and laboratory support on outbreak investigation (Center for Health Protection, 2020). It also conducts quality assurance programmes for continuous improvement of standards of laboratories in Hong Kong, and acts as a reference laboratory to provide confirmatory service, technology transfer, and training for laboratory personnel. Moreover, it collaborates with local and international partners and exchanges useful information on disease surveillance and infection control standards.
6. The **Programme Management and Professional Development Branch** provides support for the other five branches. This branch coordinates with and provides secretariat support for the Board of Scientific Advisers and various scientific committees of CHP; liaises with international and regional health authorities to facilitate collaboration activities; coordinates visits and exchange programmes with international, regional and local institutions; coordinates applied research, including research projects conducted in collaboration with universities, HA and other governmental departments; coordinates and organises professional development activities for healthcare professionals; and provides secretariat support to the Council for the AIDS Trust Fund. This branch also plans and implements various free vaccination programmes and vaccination subsidy schemes.

### CHP’s scientific advisory committees

Another important structural feature of the CHP was the formation of additional scientific advisory committees allocated to specific functions and common diseases, while working with local, regional, and international experts and health authorities (Center for Health Protection, 2009a). These seven committees release official documents and guidelines to healthcare workers and formulate thorough and effective strategies to protect public health related to their function or disease. The seven committees include:

- Scientific Committee on AIDS and STI (15 members, 2 special advisors)
- Scientific Committee on Advanced Data Analysis and Disease Modelling (5 members)
- Scientific Committee on Emerging & Zoonotic Diseases (13 members)
- Scientific Committee on Enteric Infections and Foodborne Diseases (13 members)
- Scientific Committee on Infection Control (17 members)
- Scientific Committee on Vaccine Preventable Diseases (13 members)
- Scientific Committee on Vector-borne Diseases (14 members)

**Fig. 4:**
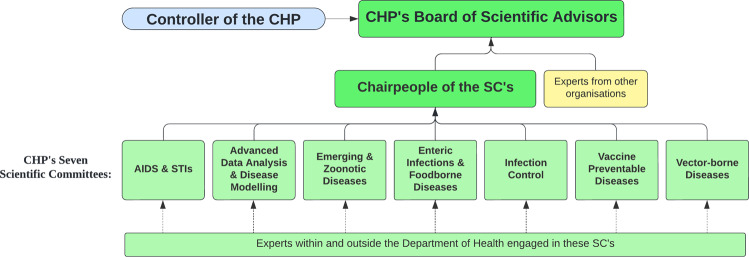
CHP’s seven scientific committees and the reporting hierarchy. The seven scientific committees operate under CHP, and each committee’s chair serves as a member on the CHP’s Board of Scientific Advisors.

CHP’s scientific advisory body comprises two organisational structure levels (see Fig. 4). On the lower, operational level, experts from various fields form these seven committees. On the higher level, the chairs of each of the seven committees, along with CHP controllers, form the CHP’s Board of Scientific Advisors (BOSA). BOSA members are appointed by the Director of Health, and serve three-year terms (Center for Health Protection, 2004b). The chairs and the CHP controllers meet regularly to ensure a broadened perspective from scientists who collectively formulate the most effective strategies that strengthen the local health protection system in Hong Kong (Center for Health Protection, 2004a).

The health organisations discussed above, as well as the science advisory institutions are summarised in Table 1, below. This includes when each was established, reasons for establishment, operational purpose, staffing numbers, background of staff, and key decision makers.

**Table 1:** Summary table of Hong Kong’s Health Organisations.

Name of Department or Committee	Date established	How/why was it established?	Main purpose	How many staff/members?	What is the general make-up of these people?	Who makes major decisions/recommendations?
Department of Health [Department of Health, 2022; Lam, 2007; Li, 2014]	April 1989	Established during Hong Kong’s public sector reform, after splitting the former Medical and Health Department into two separate departments: the Department of Health and the Hospital Services Department (which would become the Hospital Authority).	Serves as the HKSAR Government’s “health adviser and agency to execute health policies and statutory functions”. The DH safeguards the health of locals through health promotion, prevention, curative, and rehabilitative services.	4662 staff, which includes CHP’s headcount (as at March 2007)	Medical and health officers, nurses, dental, other departments and non-departmental support staff.	Four controllers who each lead Dental Services, Health Protection, Regulatory Affairs, and Health Services and Administration, report to the Director of the DH.
Hospital Authority [Hospital Authority, 2022a; 2022b; Hospital Authority Ordinance, 1997; Leong, n.d.]	December 1990	Established during Hong Kong’s public sector reform, after splitting the former Medical and Health Department into two separate departments: the Department of Health and the Hospital Services Department (which would become the Hospital Authority).	This statutory body is responsible for managing Hong Kong’s public hospital services and providing access to comprehensive, affordable, and professional preventative, curative, and rehabilitative care.	Approximately 77,000 staff (as at March 2018).	Both civil service staff and public hospital personnel, including medical doctors and officers, nurses, allied health practitioners, and administrative/support staff.	The HA is led by the Chief Executive, who oversees the management of seven public hospital clusters and eight head office divisions (e.g., Quality and Safety, Finance, Human Resources). Collectively, HA advises the Government on the needs of the public for hospital services and makes policy recommendations to the Secretary for Health.
HA Working Group on SARS [Hospital Authority, 2003; Hospital Authority Ordinance, 1997]	March 2003	Established as a specific science advisory body in response to the SARS outbreak.	This Working Group gathers senior executives from public hospitals and functional directors to compile data on SARS’s clinical definitions and features, hospital admission criteria, treatments, and environmental control, and generate guidelines, reports, and recommendations for hospital workers and support staff.	’sARS Round-up Meetings' would involve ~40 senior executives from various functions. They convened almost daily during the epidemic’s peak in March and April 2003.	Chief Executives of HA, the seven public hospital clusters, the Directors and Senior Executive Managers of various functions (e.g., cross hospital coordinator, infection control, supplies and environmental control).	The SARS Round-up Meetings was chaired by HA’s Chief Executive, while recommendations were made collectively as a Working Group.
SARS Expert Committee [HKSAR Government, 2003c; 2003d]	May 2003	Appointed by the Chief Executive after the SARS outbreak peaked in Hong Kong.	To "identify lessons to be learnt [on SARS] and make recommendations on improvement measures" such as infection controls, isolation facilities, and hospital management.	Two Co-Chairs and nine committee members.	One Co-Chair is Chairman of the Board of a prominent children’s hospital in the UK, and the other Co-Chair is the President of the Faculty of Public Health Medicine of the Royal Colleges of Physicians in the UK. The remaining nine members comprise a combination of physicians, epidemiologists, public health experts, and professors from the UK, US, Australia and Mainland China.	The Committee collectively generated a 18-chapter report as their core output, addressed to the Chief Executive.
Center for Health Protection [Center for Health Protection, 2004c; 2005; 2019]	June 2004	Established after an in-depth review of DH and Hong Kong’s health protection system, and after recommendation by the investigative SARS Expert Committee. One of Hong Kong’s largest charitable organisations, the Hong Kong Jockey Club, pledged to contribute $500 million Hong Kong dollars (~$390 million U.S. dollars) to set up CHP initially.	Summarised by 3 'R’s: Real-time surveillance, rapid intervention, responsive risk communication. In the event of an outbreak, it aims to manage timely and effective risk communications, deliver prompt responses, and activate surge capacity in collaboration with other stakeholders, including frontline staff.	"Around 1500" staff at time of establishment	The general staff are presumably trained in public health, community health, and communicable and infectious diseases. The Director of Health, the Controller, and the six branches' Heads, are all medically qualified doctors (i.e., with MBBS or equivalent) with specialties in public health, community medicine, and pathology.	The Controller
CHP’s Scientific Committees [Center for Health Protection, 2004a; 2004b]	June 2004	Established under the CHP at the outset.	As a platform for "deliberation and professional exchange among experts, upon which strategies and actions for communicable disease prevention and control can be formulated."	Each of the seven Committees had between 8–15 members, including the chairperson, totalling 74 Committee members.	The majority of each Committee’s members were medically trained doctors, while six of the seven Committees' chairperson were professors at Hong Kong medical school departments.	Each Committee’s chairperson represents their committee by being a member of the Board of Scientific Advisors, which is chaired by and reports to the Controller of the CHP.
Four workgroups under Chief Executive’s Steering Committee [HKSAR Government, 2020b]	January 2020	Established by the Chief Executive within 1 month of COVID-19’s being first discovered.	Collectively, the workgroups will help to devise relevant strategies and measures to respond to the developments of COVID-19, including liaising with the Mainland Chinese central government, WHO, local health departments, and communities.	Membership of each workgroup unknown.	Each of the four workgroups is led by the Secretaries of various departments, i.e., the top officials within the HKSAR Government.	Along with the expert advisory group, these four workgroups' leaders report to Steering Committee cum Command Centre, which is chaired by the Chief Executive.
This 8th department is fine as it is: Informal expert advisory panel	January 2020	Established by the Chief Executive within 1 month of COVID-19’s being first discovered.	To offer their expertise and advise to senior officials including the Chief Executive, regarding COVID-19’s virology and transmission mechanisms, epidemiology, social distancing and preventative measures, etc.	Four.	All four experts are professors and directors/chairs of public health and microbiology departments at Hong Kong’s two medical schools. These researchers and clinicians were pivotal in discovering the virology of SARS and investigating its clinical implications and treatments.	Meetings with the Chief Executive were scheduled as and when needed (e.g., during cluster outbreaks, new variants, the need for social distancing measures etc). Each expert also individually expressed their own analyses and opinions via various media channels, under their own authority.

## Hong Kong’s science advisory mechanism during the COVID-19 pandemic

In swift response to the emergence of COVID-19, on 4 January 2020, the HKSAR government launched the Preparedness and Response Plan for Novel Infectious Disease of Public Health Significance (“the Plan”; HKSAR Government, 2020c). The Plan lists out the strategies and government structures that would be implemented in the case of an outbreak of a novel infectious disease. The Plan also details a three-tier response system with different guidelines, protocols and resource coordination corresponding to the tier; along with launching the Plan, the government activated the’serious’ response level, the middle tier between ‘alert’ and ‘emergency’. The six areas to be addressed during an outbreak include: enhancing mechanism and organisational structure to tackle disease; strengthening immigration control; minimising risks of virus infection and spreading in local community; enhancing personal hygiene of the public; improving anti-epidemic facilities and services; and allocating sufficient resources for the strategies and measures (HKSAR Government, 2020b).

**Fig. 5:**
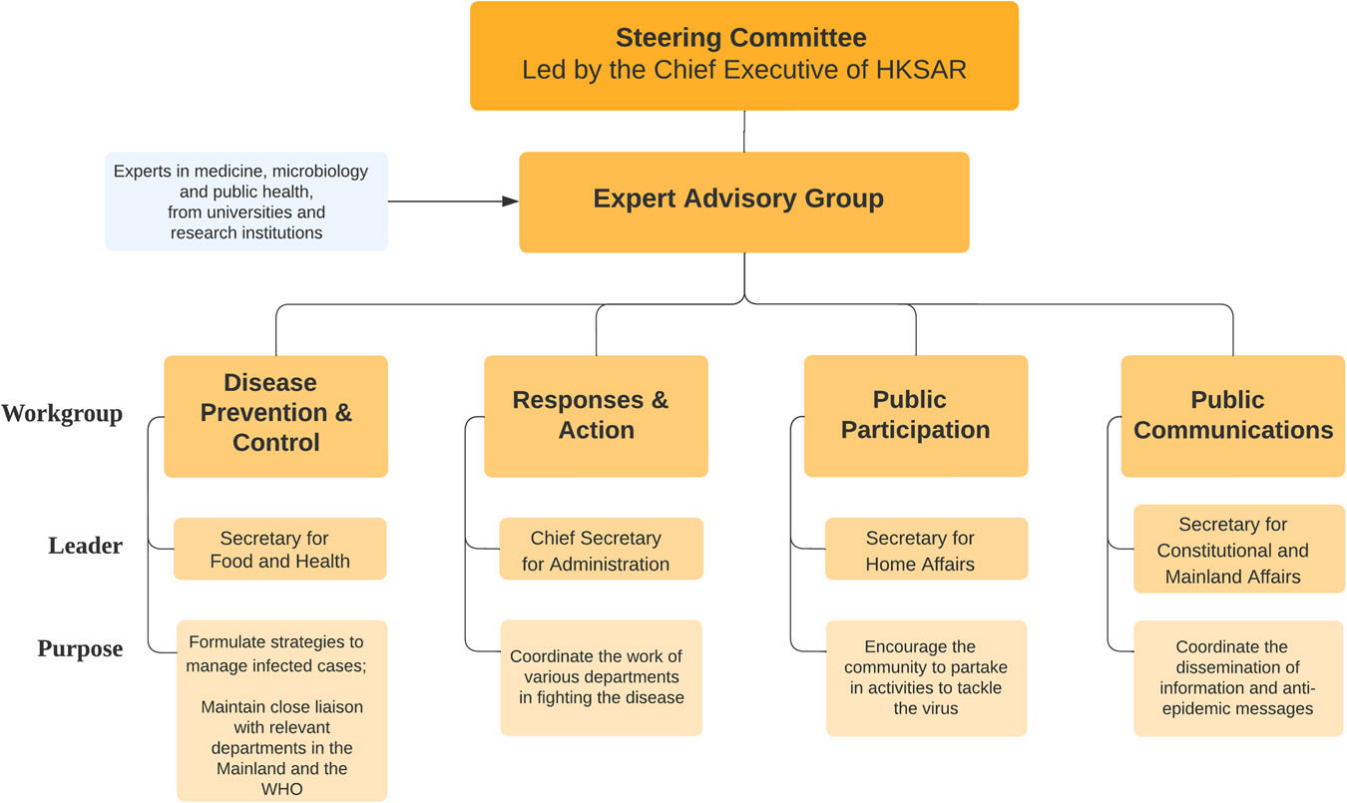
The four ‘workgroups’ under the Chief Executive’s Steering Committee. Four formal workgroups along with an informal expert advisory group report to the steering committee and command centre, created and led by the Chief Executive.

In accordance to the Plan, a steering committee and command centre were created and led by the Chief Executive to devise relevant strategies and measures in response to the developments of COVID-19 in the shortest amount of time (HKSAR Government, 2020e; see Supplementary Information Appendix 4 for core members). Under the steering committee and command centre, four formal workgroups operate with their roles as follows (see Fig. 5):

- The **Workgroup on Disease Prevention and Control**, led by the Secretary for Food and Health, is responsible for formulating strategies to manage infection cases and maintaining close liaison with relevant departments in Mainland China and WHO.
- The **Workgroup on Responses and Actions**, led by the Chief Secretary for Administration, coordinates the responses and efforts of various departments in fighting the outbreak.
- The **Workgroup on Public Participation**, led by the Secretary for Home Affairs, encourages the community to take part in activities and behaviours to tackle the virus.
- The **Workgroup on Communications** led by the Secretary for Constitutional and Mainland Affairs, ensures that the most updated and accurate information is conveyed to all members of the public and stakeholders swiftly and effectively.

If the response level is raised to the ‘emergency’ level, the steering committee could invite other senior officers or non-government experts to participate in discussions. Moreover, new sub-committee groups could be formed to manage operational matters and make recommendations to the steering committee and representatives from the DH and HA. Members joining these sub-committees could be non-government experts from relevant bureaus and departments, invited by the Secretary for Food and Health as he or she sees fit. (HKSAR Government, 2020e).

After the Plan’s response level was raised to ‘emergency’ on 25 January 2020, an *informal* expert advisory group was also announced to be set up under the steering committee and command centre, and consists of experts in various medical fields, including public health, infectious disease and respiratory medicine (HKSAR Government, 2020b). These experts were experienced in working closely with a wide range of stakeholders, including universities (as researchers and professors), healthcare workers (as physicians), international agencies (e.g., WHO, World Bank), as well as government officials (HKUMed, 2022). They were instrumental in dealing with previous outbreaks including SARS in 2003, Swine Flu in 2010 and MERS in 2012 (HKSAR Government, 2020e). The expert advisory group was appointed directly by the then Chief Executive Carrie Lam, and consists of:

- **Professor Keiji FUKUDA**, Director and Clinical Professor of the School of Public Health at the University of Hong Kong, and former Assistant Director General of the WHO;
- **Professor David HUI**, Professor of Respiratory Medicine and Director of Stanley Ho Centre for Emerging Infectious Diseases at the Chinese University of Hong Kong;
- **Professor Gabriel LEUNG**, Dean of Li Ka Shing Faculty of Medicine at the University of Hong Kong and ex-Director of the Chief Executive’s Office;
- **Professor YUEN Kwok-yung**, Chair of Infectious Diseases, Department of Microbiology, Li Ka Shing Faculty of Medicine at the University of Hong Kong.

The accomplishments of these scientists highlight the importance of past experience and the maintained reputation of these science advisors. For example, Yuen’s pivotal role in the successful discovery of the SARS coronavirus in 2002–2003 made him a go-to expert for understanding the virology of COVID-19 (HKUMed, 2018). Similarly, Hui’s experience as a frontline clinical investigator during SARS makes him a useful expert in understanding COVID-19’s clinical implications and its treatment and clinical management (Stanley Ho Centre for Emerging Infectious Diseases, 2021). As such, these experts’ scientific advice and opinions were highly accepted by the government and the public in the onset of the pandemic.

The multiple roles played by these experts as part of Hong Kong’s science advisory structures shine a light on the need for non-institutional science advice from individual experts. The credibility of these individual experts—whether they sit on scientific committees or informally appointed by the government—made them useful and effective in lending their knowledge, experience and, in some cases, their reputation, to advise the policy decision makers. Moreover, their scientific impartiality played a significant role as mediators between the government and the public amidst the ‘political new normal’ characterised by historically low public trust and high ideological polarisation (張嘉敏, 2020).

Indeed, many of these individual experts have long-standing relationships with the government and civil service, and can serve to strengthen the viability of any science-led policies while being politically informed (HKUMed, 2022). For example, given Hong Kong’s political backdrop in 2020, the informally appointed expert panel inadvertently adopted ‘extrascientific’, and sometimes political, roles, such as educating the public and moderating their emotions through the media, becoming the spokesperson or scapegoat for the government, and even criticising the government’s policies publicly (Yahoo! News, 2020). The politicisation of these experts raises new questions regarding the solidity and nature of Hong Kong’s science advisory structures and how these may evolve into the future. Chief among these is whether the relationship between experts and government will remain opaque, and even more crucially, the degree to which experts are become more or less involved in public-facing ‘extrascientific’ roles. The composition, characteristics and capabilities of the experts form the backbone of the science advisory structures and, therefore, anchors the foundation for success of pandemic response.

### The scientific advisory mechanism for COVID-19 vaccinations (late December 2020)

One of the seven scientific committees formed focuses on vaccine preventable diseases, as the CHP believes that vaccination is an important measure to protect the public from infectious diseases (Center for Health Protection, 2017). This committee scrutinises scientific evidence to provide recommendations and strategies on vaccine selection and distribution at the population level. Towards the end of 2020, the importance of vaccines in curbing COVID-19 had become increasingly apparent. Given the successful and expedient research and development of new vaccines targeting the SARS-CoV-2 virus, the Hong Kong government had created a temporary advisory panel on COVID-19 vaccines (HKSAR Government, 2020g). In their first meeting on 30 December 2020, the Chief Executive appointed experts from hospitals, universities and the DH onto the’Advisory Panel on COVID-19 Vaccinations’ (see Supplementary Information Appendix 5 for a list of experts, their fields and university affiliations). Notably, this panel comprises experts from many different medical fields, including epidemiology, paediatrics, geriatrics and pharmacology, among others, highlighting the multidisciplinary considerations of vaccine deployment while taking into account the special needs of various populations. Along with the panel, the Scientific Committee on Emerging and Zoonotic Diseases and the Scientific Committee on Vaccine Preventable Diseases both reviewed the latest global scientific evidence on epidemiology and published relevant data from overseas. This helped the panel prepare for the COVID-19 vaccination rollout that was expected to begin mid-February of 2021 (HKSAR Government, 2020a).

Even though formal scientific advisory mechanisms and informal expert appointments were functionally in place, in reality, many policy proposals and decisions on public health measures were met with widespread criticism and reluctance amid Hong Kong’s ‘political new normal’ characterised by historically low public trust and political legitimacy (Hartley and Jarvis, 2020). For example, despite the rigorous advisory panel in place for evaluating the safety and efficacy of emerging vaccines, widespread vaccine hesitancy was observed in the population, posing as a direct challenge to top-down governmental structures and ambitions (Wang et al., 2021; Luk et al., 2021). Similarly, when the government launched its ‘Universal Community Testing Programme’ in late 2020 to test all of its 7.5 million citizens over a period of 2 weeks, only 1.78 million people (~24% of the population) participated (News.gov.hk, 2020). In such cases of public defiance, their statements indicate a trend towards government shifting the burden of public communications to the informally appointed scientific experts, who seemingly had more public trust than government officials did at the time. These challenges highlight the importance of public acceptance and trust as mediating factors for successful anti-epidemic policy implementation despite the existence of robust advisory mechanisms and credible experts.

## Comparison between the responses to SARS and COVID-19 and related science advisory mechanisms

The processes adopted for tackling SARS in 2003 involved a four-pronged strategy to contain the outbreak, including early detection, swift contact tracing, prompt isolation and quarantine and effective containment (HKSAR Government, 2003b). In May 2003, three temporary committees were established by senior officials to support ongoing measures as the epidemic slowly subsided. The first committee was responsible for the disinfection drives and environmental improvements in public housing estates; the second implemented programmes to revive Hong Kong’s economy (particular in the areas of tourism, trade and employment); and the third committee promoted community engagements to “improve the physical, social and economic environments of the city” (Lee, 2003). Compared to the Plan for COVID-19, similar structures were used for both crises, where the government in 2020 adopted a decision-making structure with different working groups and a steering committee to oversee them. However, the functions of the workgroups differed in terms of the focus areas managed by each workgroup.

For example, during SARS, one of the workgroups focused on keeping the city’s economy afloat and led to the implementation of supportive policies such as the Closer Economic Partnership Agreement (CEPA; Ip, 2003), a free-trade deal designed to help Hong Kong weather its economic slump (Sun, 2017). Moreover, the’Checklist of Measures to Combat SARS’ (HKSAR Government, 2003a) stipulated that, at the highest ‘Level 2 Outbreak’, the steering committee will “assess the socio-economic impact of the crisis on Hong Kong and make decisions on the measures to minimise the impact”. At the time, economic relief packages addressed specific areas of needs, ranging from waiving water and sewage tariffs and relief of grand loans, to creating new jobs and providing vocational training for those who lost their jobs due to SARS. However, for COVID-19, the government took a broader, more comprehensive approach towards economic relief through the ‘Anti-Epidemic Fund’, including industry rent relief, business wage subsidies, unemployment support schemes and multiple rounds of consumption vouchers (HKSAR Government, 2022; see Supplementary Information Appendix 6 for government responses to COVID-19).

The second difference between the responses to the two outbreaks was the *amount of time* it took the government to take action to tackle the two epidemics. During SARS, the first workgroup named “Working Group on Severe Community-Acquired Pneumonia (SCAP)” was created on 13 February 2003, which was 1 month after the first confirmed SARS cases in Hong Kong. Another forty days later, on March 25, the government created a steering committee to coordinate the government’s resources and measures to control the epidemic that had already spread to other countries too (HKSAR Government, 2005b). In contrast, the first official account of COVID-19 cases was reported on 27 December 2019, yet the Hong Kong government launched the Plan only eight days later on 4 January 2020, announcing their decision-making structure and preparation for the novel coronavirus outbreak, even before the disease was named COVID-19 (HKSAR Government, 2020c). This structure of decision-making power involved a steering committee, the science advisory team and four working groups to tackle different aspects of the outbreak.

Some scholars have concluded that the delayed response to SARS was due to poor communication between the secretary levels of each government bureau with the management levels of the hospitals, which delayed the implementation of measures and hampered decision-making processes (Lee, 2003). Indeed, the final report by the SARS Expert Committee (2003a) recommended having one single authoritative body who would have the responsibility, authority and accountability for the prevention and control of communicable diseases. Moreover, the same report also highlighted Hong Kong’s lack of communication with Mainland China: The Guangdong Province of Mainland China first published its investigative report on 23 January 2003 about the emerging atypical pneumonia (i.e., soon to be called as SARS), but the report was only accessible to a limited number of people, none of which included any Hong Kong authorities.

During SARS, many frontline healthcare workers fell ill, while some passed away, prompting the HA to issue its first set of guidelines 19 days after the first local SARS cases (SARS Expert Committee, 2003a). These guidelines instructed hospital departments to follow certain infectious disease protocols. The need for such guidelines was also prompted by the government’s frequent, but confusing, updates on the protocols for public healthcare and private sector medical staff (SARS Expert Committee, 2003c). In contrast, ever since the COVID-19 outbreak in Wuhan, the DH, HA and permanent science advisory teams closely monitored and communicated the progress of the virus; since January 2020, the Scientific Committee on Emerging and Zoonotic Diseases released a total of six sets of consensus recommendations on the prevention and control of COVID-19 over the course of the first 7 months of the pandemic (Center for Health Protection, 2022). The strengthening and various updates of the guidelines for medical staff and the public provided clear instructions on what actions to take to prevent and slow down the spread of the virus. However, similar to the case in SARS, the government had occasionally sent mixed messages and caused confusion among the general public; for example, on 4 February 2020, the Chief Executive explained that masks were not necessary, and even ordered government officials not to wear masks “except in limited circumstances” (Chung, 2020). Five months later, the government mandated all residents to wear masks in all indoor and outdoor public areas (HKSAR Government, 2020f).

## Analysis of the differences between scientific advisory mechanisms for SARS and COVID-19

One of the fundamental reasons for the differences between the scientific advisory mechanisms during SARS and COVID-19 was the contrast in virology and epidemiology of the SARS and COVID-19 viruses and epidemics (Petrosillo et al., 2020). Owing to the SARS virus’s rapid onset and high severity of symptoms, immediate hospital admission and close monitoring of patients were required as soon as they were diagnosed (Anderson et al., 2004). This meant that decisions and protocols were largely led by specialists in medicine, especially in respiratory and critical care medicine. HA, together with the public hospitals it managed, formed the main network for SARS response. In addition, the SARS epidemic was characterised by numerous episodes of cluster infections caused by one or a few “super-spreaders”, and therefore the main strategy for controlling the epidemic was largely based on identifying and isolating the “super-spreaders” and their close contacts (Dye and Gay, 2003; Galvani and May, 2005). Thus, during the SARS epidemic, science advice was predominantly offered for public health and medical decisions, rather than for other aspects like the public’s social life or the economy.

In contrast, the virology and epidemiology of COVID-19 were significantly different from SARS. First, the first variants of the COVID-19 virus had a slower onset than SARS, with an 80% milder disease rate, meaning that most patients exhibited mild, or even no symptoms (Liu et al., 2020). Additionally, although there were few “super-spreaders,” the social activities of large numbers of infected, but asymptomatic, individuals tended to increase the *scale* of cluster infections geometrically (Lakdawala and Menachery, 2021). Here, the focus of governmental response was not confined only to the medical field: Having assumed that this less deadly, but more infectious, virus would persist for a long period of time, the government focused more resources on testing capacity, vaccine application and everyday epidemic prevention, such as capacity enhancement for the treatment of mild patients, control of individual movement and social activities, and travel and immigration controls (Hale et al., 2022). This demonstrates an important ability to adapt crisis plans to the particulars of the disease driving the pandemic, as opposed to being stuck responding as if to the earlier crisis.

During SARS, the lack of established governmental organisations had slowed down the exchange of epidemiological information between health authorities in Mainland China and Hong Kong (Lee, 2003), and subsequently the speed of implementing the strategies designed to protect frontline healthcare workers who were most at risk. The SARS epidemic triggered the creation of the CHP by the DH, and one of the key subsequent developments was the regional coordination between the local Hong Kong and national Mainland healthcare systems: In October 2005, Hong Kong, Macau and Mainland China signed a joint “Agreement on emergency response mechanism for public health emergencies” (the “Cooperation Agreement”), which sought to strengthen the cross-border links between the three health care systems (HKSAR Government, 2005b). This agreement was signed as the trio of governments anticipated that rapid globalisation would lead to public health crises spreading across borders in the future. This agreement intended to speed up the coordination and mobilisation of manpower, techniques and resources between experts from the three places, and expedite the creation of joint crisis response teams to devise response strategies for situations like the Swine Flu in Sichuan or SARS (Center for Health Protection, 2009b). This agreement had also made cross-border initiatives easier, including the transfer of Hong Kong residents from Wuhan to Hong Kong in 2020 and allowing Hong Kong residents in Shenzhen to obtain medicines from Chinese hospitals. The maintenance of an open line of communication vertically between various leadership and operational levels and horizontally between different territories remains an important element to achieve swift pandemic response coordination and avoid an institutional crisis during any future pandemics.

Technology has advanced considerably between 2003 and 2020, and the Innovation and Technology Bureau took advantage of the widespread use of the internet to disseminate information, announcements and guidance to the public swiftly, as well as tracing close contacts with mobile applications and distributing copper-lined reusable face masks for each Hong Kong citizen for free (Innovation and Technology Bureau, 2020). For example, in 2020, for a temporary hospital built within one of Hong Kong’s convention centres called AsiaWorld-Expo, HA created an e-health system to enable COVID-19 patients to self-monitor and transmit vital signs to health monitors through a mobile app. This reduced unnecessary social interactions and ensured potential transmissions to be identified and isolated swiftly (HKSAR Government, 2020d). The indisputable value of technological innovations in pandemic mitigation and therapeutics (e.g., vaccinations, rapid testing, and clinical drugs) calls for increased governmental and private investments in such technologies, in preparation for future pandemics and public health challenges (Neville and Kuchler, 2022).

While pandemic response for COVID-19 had certainly benefited from lessons learned during SARS, these initial benefits eventually faced new emerging challenges. Moving into 2021 and 2022, vaccination hesitancy—due to the persisting low trust in government, and by proxy, low trust in scientific experts—continued to dampen vaccination rates, particularly among the elderly population who eventually fell most victim to Hong Kong’s deadly fifth wave in early 2022 (Cheung et al., 2022). Increasingly opaque decision-making, unpredictable policy directions and frequent policy U-turns continued to frustrate numerous segments of the population with stringent policies such as school closures, tight social distancing measures and travel restrictions (Ho, 2022). During its fifth wave, which was driven by the milder but much more transmissible Omicron variant, Hong Kong’s public health institutions and hospitals were overwhelmed and pushed to its limits when it logged the world’s highest number of deaths per population, as mortuaries became full and deceased bodies were placed in refrigerated freight containers and inside hospital wards next to inpatients. (Taylor, 2022; Cheng, 2022). As the situational and institutional crises unfolded rapidly in real-time, Hong Kong sought scientific advice and operational assistance from Mainland China, who sent down hundreds of volunteer medics, built temporary quarantine facilities, donated millions of traditional Chinese medicine packets as treatment and appointed a new team of mainland experts (McGregor, 2022). Having fortunately ridden this turbulent wave, which lasted 3 months, Hong Kong faces yet another major institutional quandary. Will Hong Kong comply with China’s insistent national ‘zero-COVID policy’, or will it digress from the central government’s strategy, ‘live with COVID’ and prepare for a full revitalisation of its economy (Dyer, 2022)? More specifically, will Hong Kong’s recent ‘failures’ during the fifth wave discredit its own science advisory structures and experts’ credibility, and eventually compel the local government to embrace a public health system that is much more unified with the central government?

## Conclusion

Hong Kong’s response to the COVID-19 pandemic had been directly shaped by its experience with SARS in 2003. In January 2020, as the scale of the pandemic emerged, the Hong Kong SAR Government was able to leverage on a series of existing policies, structures and advisory groups. The expert working groups established under the name of HA during SARS was the predecessor, while the CHP, established under DH on the basis of the reflection on SARS, laid the foundation of the scientific advisory system in place in 2020. Since the establishment of CHP, Hong Kong had eventually formed a scientific advisory mechanism led by government departments and involving experts from various organisations and fields to respond to public health incidents.

From the SARS to the post-SARS period, and then during the COVID-19 period in 2020, the scientific advisory mechanism had become progressively more complex and flexible. Specifically, during the SARS and post-SARS periods, the scientific advisory mechanism mainly included officials from DH and HA as well as experts in the fields of medicine (especially respiratory and critical care medicine), pharmacology and public health. In the response to COVID-19, it had involved experts from broader and more fields, in order to adapt to the characteristics of this new epidemic. However, the range of expertise had not extended to areas such as economics, mental health or social welfare, and retained a bias towards different areas of public health and medicine. Moreover, during COVID-19, the scientific advisory mechanism was no longer limited to CHP, DH and HA. The government had expanded its organisational structure to enhance interdisciplinary collaboration with other government departments under the framework of the Steering Committee.

In addition to the use of these formalised advisory structures, the government had also relied extensively on well-respected local experts as a key part of its public communications strategy and receiving science advice. Three experts, in particular, had visibly communicated their science advice and expert opinions through various public-facing media channels, such as radio talk shows, televised programmes and press conferences, with much of Hong Kong’s population paying attention. With only medical experts in the government’s expert advisory panel, the panel’s specialty was somewhat confined to science advice, while there was a lack of diversity in terms of other potential experts who could advise more appropriately on, for example, the economy, schools and industry, all of which were greatly affected by the COVID-19 pandemic.

Our full understanding of the roles of science advisory groups and experts in actual decision making still remains limited. The number of formal documents and statements issued by the advisory committees has been quite scant (see Supplementary Information Appendix 7 for information on formal documents) and transparency around the contents, or even schedules, of the meetings and interactions with key decision makers was low. Even the process through which experts were identified and appointed remains unclear. More research, such as interviews with specific science advisors, is required to develop a fuller, behind-the-scenes picture of how science advice was developed and communicated to decision makers, as well as the public. Furthermore, this research highlights the importance of understanding public trust in government, scientific experts and other key civic leaders, and this requires more extensive research in various political environments similar and dissimilar to Hong Kong in 2020, in order to better understand the strengths and weaknesses of the science advisory systems in place.

A pandemic can indeed be defined theoretically as a crisis. Based on Rosenthal and Kouzmin (1997), the COVID-19 pandemic posed as an urgent threat to Hong Kong’s healthcare system, economy, livelihoods and its usual sense of safety and unity, and with each subsequent outbreak, policy-makers were required to make swift and vital decisions under high uncertainty and time pressure, but also immense *public* and *political* pressure. While expert science advice may help to avert a pandemic’s situational crisis, it may not necessarily prevent the onset of an institutional crisis, which could be fuelled by internal factors like politics and poor inter-agency communication, as well as external factors like low public trust in government and a highly polarised, defiant public. In practice, a pandemic can also be framed as an invaluable opportunity for policy learning and for strengthening crisis leadership. As Hong Kong enters the recovery phase of the crisis management cycle, it must reflect carefully on and learn from its successes as well as its mistakes and failures, lest it stays unprepared for the next potential wave or the next pandemic. Just as COVID-19 had learned lessons from SARS, will our next pandemic have learned lessons from COVID-19?

## Supplementary information

Supplementary Material

## Data Availability

Data sharing is not applicable to this research as no data were generated or analysed.
