# Supplementary material for: From SARS to COVID-19: the role of experience and experts in Hong Kong’s initial policy response to an emerging pandemic
Source: Humanit Soc Sci Commun. 2023 Jan 5;10(1):9. doi: 10.1057/s41599-022-01467-z (PMC9815065; doi:10.1057/s41599-022-01467-z)
Supplement: Supplementary file 1 — Supplementary Material [file 41599_2022_1467_MOESM1_ESM.docx]

# Supplementary Information Appendix 1 – COVID-19 Case Data for 2020 in Hong Kong SAR (Data from data.gov.hk)

**
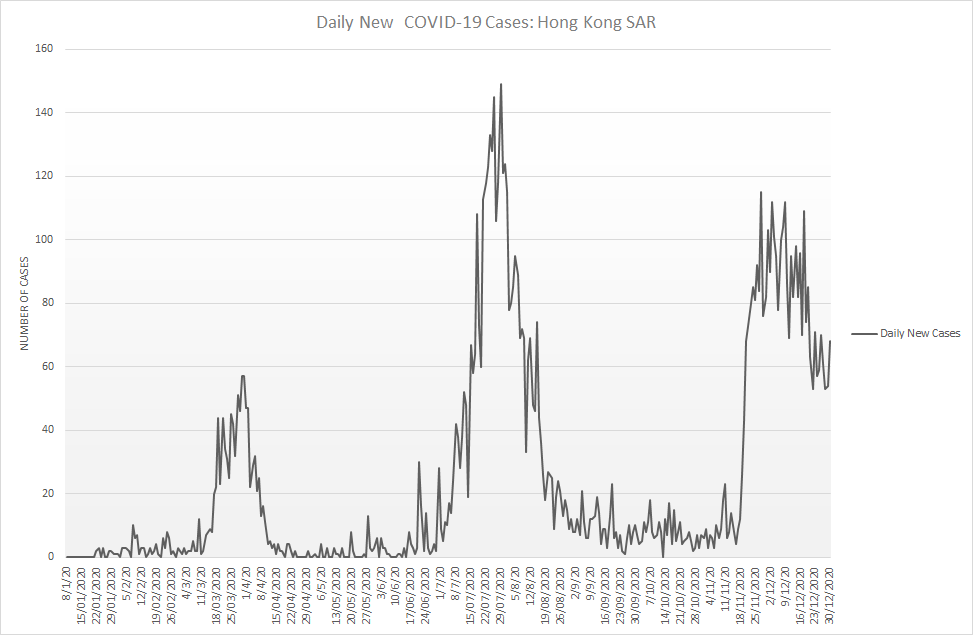
**


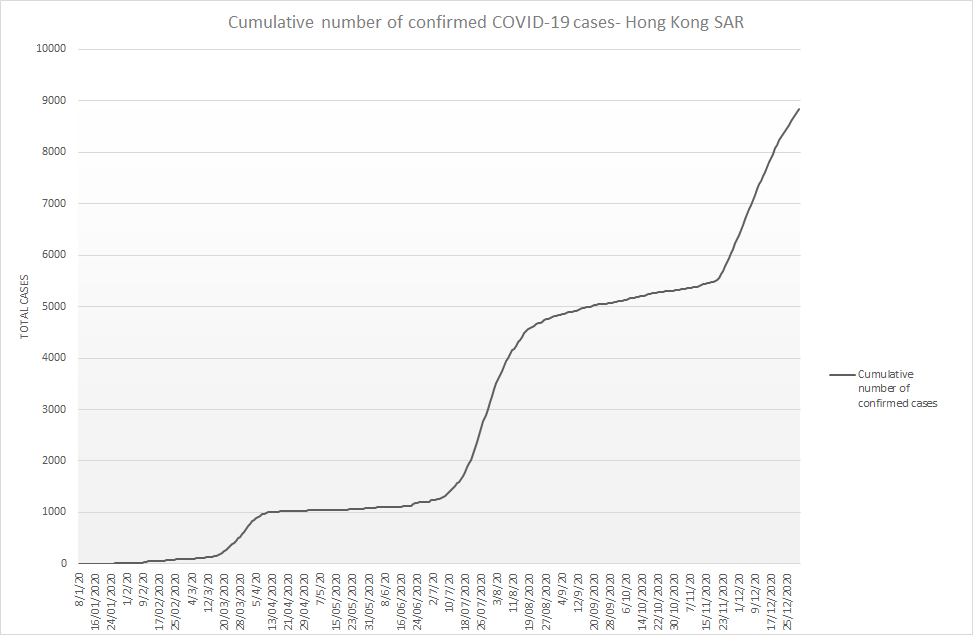


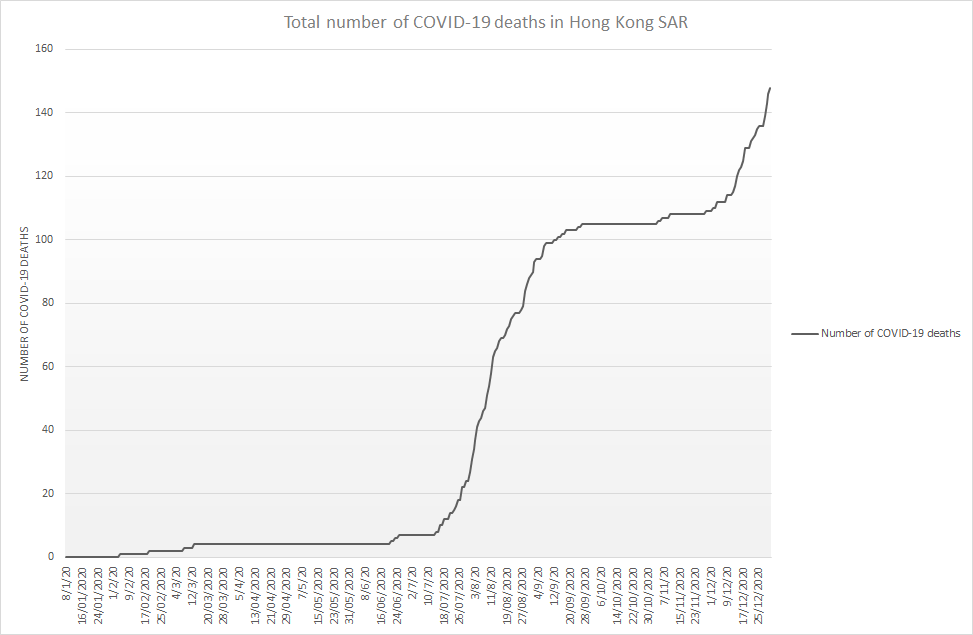


# Supplementary Information Appendix 2 – SARS Expert Committee to review on SARS in post-SARS era

**
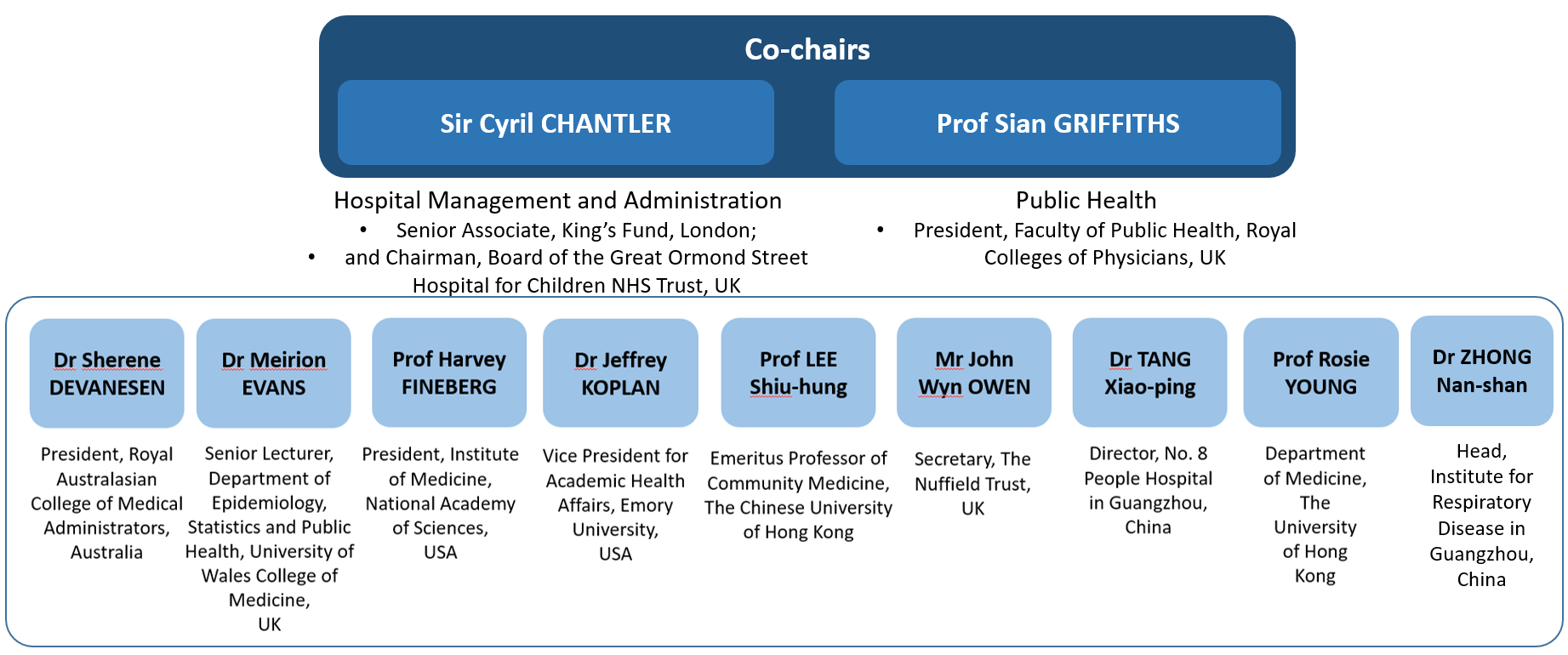
**

Co-chairs:

- Sir Cyril CHANTLER (Hospital Management and Administration), Senior Associate, King’s Fund, London; and Chairman, Board of the Great Ormond Street Hospital for Children NHS Trust, UK
- Prof Sian GRIFFITHS (Public Health), President, Faculty of Public Health, Royal Colleges of Physicians, UK

Members:

- Dr Sherene DEVANESEN, President, Royal Australasian College of Medical Administrators, Australia
- Dr Meirion EVANS, Senior Lecturer, Department of Epidemiology, Statistics and Public Health, University of Wales College of Medicine, UK
- Prof Harvey FINEBERG, President, Institute of Medicine, National Academy of Sciences, USA
- Dr Jeffrey KOPLAN, Vice President for Academic Health Affairs, Emory University, USA
- Prof LEE Shiu-hung, Emeritus Professor of Community Medicine, The Chinese University of Hong Kong
- Mr John Wyn OWEN, Secretary, The Nuffield Trust, UK
- Dr TANG Xiao-ping, Director, No. 8 People Hospital in Guangzhou, China
- Prof Rosie YOUNG, Department of Medicine, The University of Hong Kong
- Prof ZHONG Nan-shan, Head, Institute for Respiratory Disease in Guangzhou, China.

# Supplementary Information Appendix 3 – Drills Conducted by the CHP to Improve Preparedness

Drills conducted by the CHP’s ERIB since 2010 (Centre of Health and Protection, 2019):

- 2010, June 3 & 4 - joint practice with DH and Airport authority named “HUA SHAN” which mimics an outbound flight returned to Hong Kong because a passenger was potentially carrying a severe infectious disease. Government departments and relevant organizations collaboration required reactions towards the incident
- 2010, October 25 – project NEPHRITE was done to test relevant departments’ responses to an imported plague
- 2011, May 9 – JASPER was conducted by CHP, FG and Shenzhen Entry-Exit Inspection and Quarantine Bureau
- 2012, January 9 - Jadeite was held to test the preparedness of government departments and relevant organizations to respond effectively in screening and evacuating involved residents in a major infectious disease incident. The exercise provided an opportunity for the DH and relevant organizations to try out relevant contingency plans and identify areas for improvement. It also enhanced community and healthcare personnel awareness, preparedness and the ability to detect and respond to possible epidemics
- 2013, January 17 and 25 - Exercise “AMBER” was held on The Exercise simulated that three inmates of an elderly home were close contacts of a novel influenza. On-site quarantine was set up in the elderly home where the three inmates stayed. The Exercise aimed at testing the response actions to the locally acquired novel influenza infection of a Hong Kong citizen, with emphasis on the raising of the response level according to the risk to the community.
- 2013, March 27: The CHP arranged a high-level interdepartmental desktop exercise code-named Exercise “RUBY” for testing preparedness for the possible outbreaks of Severe Respiratory Disease associated with Novel Coronavirus. The exercise had served to formulate and oversee the implementation of a coordinated and timely response and disease control strategy; and decide on measures to be taken which will have a wider impact on the community.
- 2013, November 21 & 2014, March 3 - Exercise “CORAL” took place where they simulated the situation where Hong Kong encountered confirmed local cases during the peak season of dengue fever. The scenario centred on the identification of a Lantau resident as being the first confirmed local dengue fever case since 2010. Response measures including vector investigation and control as well as surveillance of suspected cases were carried out immediately.
- 2014, June 23 - the CHP also held an exercise codenamed “OPAL” The exercise simulated influenza pandemic in Hong Kong, where large numbers of patients were admitted in all seven hospital-clusters under the Hospital Authority and huge demand for anti-influenza drugs were required. Various services/units under the DH activated their contingency plans, opened temporary clinics, distributed the drugs and replenished stocks by emergency procurement with the assistance of the Government Logistics Department.
- 2014, August 20 - Exercise “TOPAZ” involves a series of multi-agency drills based on the Ebola virus disease (EVD) Preparedness and Response Plan. The exercise was divided into five parts, including “Village House Disinfection” testing the co-ordination and procedures between the CHP and the Food and Environmental Hygiene Department (FEHD).

**Supplementary Information Appendix 4 - The Steering Committee and Command Centre of Hong Kong Government in response to COVID-19**

In the Steering Committee and Command Centre, the core members include:

- Permanent Secretary for Transport and Housing / Director of Housing,
- Permanent Secretary for Security, Permanent Secretary for Food and Health,
- Permanent Secretary for Transport and Housing,
- Permanent Secretary for Food and Health (Food),
- Permanent Secretary for the Environment / Director of Environmental Protection,
- Permanent Secretary for Constitutional and Mainland Affairs,
- Permanent Secretary for Education, Permanent Secretary for Commerce and Economic Development (Commerce, Industry and Tourism),
- Permanent Secretary for Development,
- Under Secretary for Food and Health, Department of Health,
- Director of Social Welfare, Department of Agriculture, Fisheries and Conversation, Department of Food and Environmental Hygiene,
- Director of Leisure and Cultural Services,
- Director of Information Services,
- Director of Home Affairs,
- Commissioner for Tourism,
- Director of Water Supplies, Commissioner for Transport, Director of Highways,
- Director of Civil Engineering and Development,
- Director of Architectural Services,
- Director of Drainage Services,
- Director of Electrical and Mechanical Services,
- Director of Lands,
- Director of the Hong Kong Observatory,
- Government Property Administrator,
- Controller of Centre for Health Protection,
- Controller of Centre for Food Safety and
- Chief Executive of Hospital Authority (HKSAR, 2020).

**Supplementary Information Appendix 5 - Advisory Panel on COVID-19 Vaccines**

|  | **Expert** | **Field** | **Affiliation** |
| --- | --- | --- | --- |
| **Convener** | **Prof Wallace Lau Chak-sing** | **Clinical immunology** | **President of the Hong Kong Academy of Medicine;**  **The University of Hong Kong** |
| **Members** | **Dr Au Yeung Tung-wai** | **Geriatrics** | **The University of Hong Kong** |
|  | **Prof Keiji Fukuda** | **Influenza epidemiology** | **The University of Hong Kong** |
|  | **Prof Lau Yu-lung** | **Pediatrics and adolescent medicine** | **The University of Hong Kong** |
|  | **Dr Ho King-man** | **Dermatology and venereal diseases** | **Fanling Integrated Treatment Centre** |
|  | **Prof David Hui Shu-cheong** | **Emerging infectious diseases** | **Chinese University of Hong Kong** |
|  | **Prof Ivan Hung Fan-ngai** | **Emerging infectious diseases** | **The University of Hong Kong** |
|  | **Prof Gabriel Matthew Leung** | **Influenza epidemiology** | **The University of Hong Kong** |
|  | **Prof Raymond Liang Hin-suen** | **Leukemia, oncology, molecular biology** | **The University of Hong Kong** |
|  | **Dr Thomas Tsang Ho-fai** | **Community medicine** | **The Department of Health, HKSAR** |
|  | **Prof Ian Wong Chi-kei** | **Pharmacology and pharmacy** | **The University of Hong Kong** |
|  | **Prof Yuen Kwok-yung** | **Microbiology** | **The University of Hong Kong** |

# Supplementary Information Appendix 6 – Timeline of COVID-19 pandemic and government responses in Hong Kong

Jan 23: First imported case of infection in Hong Kong

Jan 25: Carrie Lam announced ‘Preparedness and Response Plan for Novel Infectious Disease of Public Health Significance’ would be raised to emergency level

Jan 28: Civil servants to work from home

Jan 29: All schools are suspended and implemented public health controls

Jan 30: Semi Closing borders: Suspension of the high-speed rail service between Hong Kong and China, as well as all cross-border ferry services

Feb 4: First death from COVID-19 recorded in Hong Kong

Feb 7: Government introduced prison sentences to those who breach quarantine rules

Mar 2: Hong Kong reached 100 confirmed cases

Mar 19: Mandatory two-week quarantine extended to all arriving passengers, including residents

Mar 20: 48 new infections in one day (largest daily total so far)

Mar 25: Hong Kong announces the closure of its border to all incoming non-residents arriving from overseas. Transiting through Hong Kong was also no longer allowed. In addition, all returning residents were subject to compulsory quarantine for 14 days. Returning residents from high-risk countries were required to go through enhanced screening procedures and submit a saliva sample for testing

Mar 29: The Hong Kong government announces further restrictions, banning any indoor or outdoor gathering of more than four people. Restaurants were also required to operate at half their capacity and to set their tables at least 1.5 metres apart

Apr 3: Due to Lan Kwai Fong cluster, pubs and bars were ordered to be closed for 14 days

Apr 4: OT&P announce a study with the University of Hong Kong. The study will provide evidence of the effectiveness of the community-wide interventions that Hong Kong has been implementing. The study required 1,500 volunteers to donate a blood sample.

May 5: With no new local transmissions for more than two weeks, gyms, bars, cinemas and gaming centres reopen. Public gathering limit raised to eight

May 30: There was a confirmed case of a 34-year-old woman with no recent travel history, resulting in an extension of Hong Kong's social distancing measures

June 4: A cluster was identified in Kwai Chung and Sha Tin, Hong Kong. As a result, the Hong Kong Government evacuated some residents

June 19: Group gathering limit raised to 50 from eight. The caps on other measures are also raised

July 11: Anti-epidemic measures concerning eateries and other venues tightened

July 19: New rule mandates wearing of face masks on public transport. Ban on dining-in at all eateries from 6pm to 5am and all gatherings capped at four, while some businesses must shut for seven days

July 23: Face masks to be worn in all indoor public venues

July 27: Social distancing measures tightened further, including public gatherings to be capped at two, masks to be worn at all times in outdoor public places, and complete ban on dining-in at all eateries

Sept 1: Hong Kong government launches the universal community testing program; all Hong Kong residents can get one-off free COVID-19 testing.

Sept 4: HK Government relaxes social distancing restrictions: including restaurants can offer dine-in service till 10 pm and indoor premises can be opened (gym and massage parlours)

Sept 11: Further relax social distancing restrictions - public groups from two to four people and wearing a mask is not mandatory for outdoor exercises

Sept 18: Further relax social distancing restrictions – dine-in till midnight, indoor premises like bars karaoke and theme parks can reopen

Oct 30: Continues to ease social distance rules as COVID-19 cases decline – dine-in till 2 AM, pubs can service up to 4 people and restaurants up to 6 per table

Nov 16: Moderna claims its COVID-19 vaccine is 94.5% effective

Nov 18: Pfizer’s vaccine is 95% effective against COVID-19, according to the interim analysis

Nov 23: Hong Kong entered a fourth wave of COVID-19 outbreak with over 600 cases linked to the dance club cluster - all kindergartens and primary schools closed. Plans for the Hong Kong-Singapore travel bubble have been put on hold. Hong Kong government launched a contact tracing initiative in a mobile app format to track untraceable infections.

Dec 3: HKEB announced school closed until 2021

Dec 10: The Hong Kong Government continues to tighten social distancing restrictions to cope with the fourth wave of COVID-19 infections - Social gathering that includes more than a group of two people is banned, no dine-in services after 6:00 pm and Gym, beauty salons, amusement parks, swimming pools and other premises are also closed.

Dec 11: The Hong Kong Government has announced the procurement of three different vaccine candidates which will be dependent upon peer-reviewed evidence from ongoing studies.

#

# Supplementary Information Appendix 7 - Policy Documents

Policy Documents detailing the consensus of the committees were published on the various CHP committee websites. The memos were brief in nature, but contained important moments in the different scientific advisory’s committee understanding and policy suggestions for dealing with the virus.

| Date | Committee | Title | Brief Description |
| --- | --- | --- | --- |
| January 24, 2020 | Scientific Committee on Emerging and Zoonotic Diseases and Scientific Committee on Infection Control | Recommendations on prevention and control of novel coronavirus infection | Members have reached consensus on the following recommendations based on the prevailing local and global situation of novel coronavirus infection: mask wearing in public, community transmission, close contact on planes, mass events |
| February 17, 2020 | Scientific Committee on Emerging and Zoonotic Diseases and Scientific Committee on Infection Control | Recommendations on prevention and control of novel coronavirus infection | Evidence of community transmission of COVID-19 in Hong Kong, quarantine measures, China immigration policy, enhanced monitoring for milder cases |
| February 28, 2020 | Scientific Committee on Emerging and Zoonotic Diseases and Scientific Committee on Infection Control | Consensus on the Use of Surgical Mask by the General Public in Public Places | In this meeting, members have reached consensus on the use of surgical mask by the general public in public places |
| May 6, 2020 | Scientific Committee on Emerging and Zoonotic Diseases | Consensus Recommendations on COVID-19 and SARS-CoV-2 | Study of viral load of SARS-CoV-2 from respiratory specimens, suggestion for when to release patients |
| July 6, 2020 | Scientific Committee on Emerging and Zoonotic Diseases | Updated Consensus Recommendations on Criteria for Releasing Confirmed COVID-19 Patients from Isolation | Criteria for releasing a confirmed COVID-19 patient from isolation |
| July 29, 2020 | Scientific Committee on Emerging and Zoonotic Diseases | Updated Consensus Recommendations on Criteria for Releasing Confirmed COVID-19 Patients from Isolation | Updated criteria for releasing a confirmed COVID-19 patient from isolation |
| Jan 7, 2021 | Scientific Committee on Emerging and Zoonotic Disease and Scientific Committee on Vaccine Preventable Diseases | Consensus Interim Recommendations on the Use of COVID-19 Vaccines in Hong Kong | Provides recommendation of the use of COVID-19 vaccines in Hong Kong. |
